# Modulation of Gut Microbiome Composition and Function in Experimental Colitis Treated with Sulfasalazine

**DOI:** 10.3389/fmicb.2017.01703

**Published:** 2017-09-07

**Authors:** Haihui Zheng, Mingyi Chen, Yuan Li, Yuanyuan Wang, Lin Wei, Ziqiong Liao, Mengxia Wang, Fangli Ma, Qiongfeng Liao, Zhiyong Xie

**Affiliations:** ^1^School of Pharmaceutical Sciences, Sun Yat-sen University Guangzhou, China; ^2^School of Chinese Materia Medica, Guangzhou University of Chinese Medicine Guangzhou, China; ^3^Infinitus (China) Company Ltd. Guangzhou, China

**Keywords:** gut microbiome, 16S gene sequencing, metagenomics, colitis, TNBS, SASP

## Abstract

Inflammatory bowel disease (IBD) results from alterations in intestinal flora and the immune system. Sulfasalazine (SASP) is a sulfa antimicrobial used to treat IBD in clinic for years. However, how SASP affects gut microbes and its potential functions remains unclear. To investigate the relationships of SASP, IBD, and gut microbiome, we used 2,4,6-trinitrobenzenesulfonic acid (TNBS) to induce experimental colitis in rats, and analyzed the microbiota in the fecal samples, which come from the control group (treated with ethanol + saline), the model group (treated with TNBS-ethanol + saline) and the SASP group (treated with TNBS-ethanol + SASP), with 16S gene sequencing and followed up a subset sample using shotgun sequencing. The study found that SASP treatment could not only restore the TNBS-induced gut dysbiosis, which was proved by the increasing amount of SCFAs-producing bacteria and lactic acid-producing bacteria as well as the decreasing amount of Proteobacteria, but also modulate the dysregulated function of the TNBS-induced colitis to resemble that of the control group, including an increased capacity for basic metabolism (carbohydrate metabolism, citrate cycle) and a decrease in the oxidative stress (riboflavin, sulfur, cysteine) as well as bacterial pathogenesis (cell motility and secretion, bacterial motility proteins, flagellar assembly). Moreover, a higher proportion of *Mycoplasma* was observed in the SASP group, which may associate with infertility. In all, the study provides insight into specific microbial clades and pathways linked with SASP treatment to elaborate the mechanism for treatment of IBD.

## Introduction

Inflammatory bowel disease (IBD), including ulcerative colitis (UC) and Crohn’s disease (CD), is deemed to arise from a dysregulated immune response to gut microbial communities in individuals with genetic predisposition (Ott et al., 2004; Presley et al., 2012). Because the precise etiology of IBD still remains unknown, the management of IBD has mainly relied on palliative therapy with several non-specific agents for years, such as corticosteroids (Engel and Neurath, 2010), antibiotics (Dethlefsen et al., 2008), immunosuppressor (Pineton de Chambrun and Sandborn, 2012), synbiotics (Furrie et al., 2005), and agents aiming pro-inflammatory pathways. Nevertheless, many existing therapies are not effective for all patients and some even carry high-risk side effects or complications. Additionally, how these therapies influence the composition and function of the gut microbiome is poorly understood. Considering the pivotal role of gut microbiome in IBD, studying the altered microbe and microbial metabolism pathways linked to the host in the process of IBD treatment may help us further understand their therapeutic effects and side effects, and also can provide us improved therapeutic targets.

Sulfasalazine (SASP), which is a prodrug that is split in the colon by bacterial azoreductases to release 5-aminosalicylic acid (5-ASA) and sulfapyridine (Cooke, 1969), was developed as a drug for IBD treatment in 1940 and it also can be used in the treatment of rheumatoid arthritis. So far, SASP is still widely used in clinic, though it may cause some side effects, including nausea, anorexia, hemolysis, as well as headache (Peppercorn, 1984). Also, reversible infertility has been reported as one of the side effects that caused by SASP, but little attention has been paid on it (Toovey et al., 1981). In spite of the wide use of SASP, the concrete mechanism is still unexplained. The possible mechanism might be explained as an antibacterial effect of sulfapyridine or an antioxidant effect of 5-ASA or an effect of 5-ASA on prostaglandin metabolism (Azad Khan et al., 1977; Hazenbe et al., 1982). Considering the relationship of gut microbiome, SASP, and IBD, there is a possible way to clarify the mechanism of SASP from the perspective of gut microbiome. Some researchers have investigated the effect of SASP on the intestinal flora via culture-dependent and molecular methods. West et al. (1974) concluded that SASP treatment could give rise to a decrease of total non-sporing anaerobes, opalescent-negative clostridia, and Enterobacteria. Levitan and colleagues demonstrated an increased number of anaerobic *Lactobacilli* and Gram-positive aerobes in patients who had received SASP (Gorbach et al., 1968). Cooke (1968) discovered that there was little difference between the IBD patients with and without SASP treatment. Thus, there are conflicting and even paradoxical results based on the previous studies and it needs further verification. In addition to, the interactions between SASP and intestinal flora are still poorly explored because of the technical restriction. Fortunately, high-throughput sequencing can provide us technical support to explore 40% of intestinal flora are uncultured yet (Eckburg et al., 2005), setting a stage for the research of the IBD microbiome. In this case, we intended to explore the effects of SASP on intestinal flora to elaborate the mechanism for treatment of IBD by means of metagenomics.

In view of the human genome diversity and variability in treatments and environment, it is difficult to discern the microorganisms and metabolism pathways involved in IBD as well as the host–microbiota responses to IBD-directed therapies in human. Under the circumstances, the preclinical model of IBD is a good alternative to study the microbiome involved in IBD underlying the therapies (Peloquin and Nguyen, 2013). More than 60 experimental animal models of IBD has been established (Mizoguchi, 2012). The 2,4,6-trinitrobenzenesulfonic acid (TNBS)-induced colitis model shared most features with human CD. Thus, the TNBS-induced colitis offer an inexpensive and reproducible model for exploring the relationships of IBD, SASP, and gut microbiome.

In order to evaluate how gut microbiome contributes to the colonic inflammatory etiopathogenesis and depict the trait of gut microbiome reacting to SASP, we investigated the effects of SASP treatment on host disease status and on gut microbiome composition and function in TNBS-induced colitis model with 16S rRNA gene sequencing and shotgun metagenomic sequencing. The results demonstrated that the experimental colitis with SASP treatment could alleviate inflammation and restore the dysregulated microbiota composition and function of rats with colitis into a normal condition as the control group. In addition, the increased *Mycoplasma* in the SASP group may be used to explain why infertility would occur in those IBD patients with SASP treatment.

## Materials and Methods

### Induction of Experimental Colitis

Animal experiments were permitted and performed in strict accordance with the manuals of Institutional Animal Care and Use Committee (IACUC) of Guangzhou University of Chinese Medicine. SPF male Sprague-Dawley rats weighing between 180 and 200 g were procured from Guangdong Medical Laboratory Animal Center [Permission No: SCXK (Yue) 2013-0002] and kept under 12–12 h light–dark cycle with controlled temperature (24°C) and 50–70% humidity, they had free access to commercial rodent food and water unless special circumstances. After acclimatization for 7 days, a total of 18 rats were randomly and equally divided into three groups as follows: the control group (treated with ethanol + saline), the model group (treated with TNBS-ethanol + saline), and the SASP group (treated with TNBS-ethanol + SASP). The TNBS-induced colitis were established based on the recognized IBD model by Morris et al. (1989). To induce the colitis, rats were fasted for 24 h and then lightly anesthetized with 2% pentobarbital sodium (0.1 ml/100 g) through intraperitoneal (ip) injection and TNBS (40 mg/kg in 50% ethanol) was rectal administered once a week for 2 weeks. The rats in the control group were treated with 50% ethanol enema alone, and the rats in the model group and the SASP group receiving TNBS-50% ethanol enema. After 2 weeks’ TNBS administration, rats in the control group and the model group were subjected to gavage with normal saline by equal volume of SASP while the SASP group were orally administered at a dose of 300 mg/kg/day for 16 consecutive days, respectively. During the experiment, weight, stool consistency, and the presence of blood at the anus and in stools were observed daily. After drug treatment, stool samples and serum were collected and all rats were dissected to obtain colon tissue. The lengths of colon tissues were record and then were fixed with 10% neutral buffered formalin for histopathological observation.

### Assessment of Colitis

Blood samples were centrifuged at 3000 rpm for 5 min. The serum myeloperoxidase (MPO) level was measured by using the rat MPO ELISA kit with the manufacturer’s instructions. Changes in absorbance at 450 nm were measured by a spectrophotometer.

After fixation for 48 h by 10% neutral-buffered formalin, the colons were embedded in paraffin wax according to routine procedures (Rubin and Baur, 1983). 5 mm thick sections were cut and stained with hematoxylin–eosin for histopathological evaluation.

Symptomatic parameters (body weight and severity of diarrhea) were observed and recorded daily during the experimental period. Stool for occult blood was tested with colloidal gold method using fecal occult blood test kit. A disease activity index (DAI) was determined by scoring the extent of body weight loss, stool consistency, and stool occult blood positivity, or gross bleeding in accordance with the method described by Murthy et al. (1993) (see Supplementary Table S1).

### DNA Extraction

Fecal samples were collected in Eppendorf tubes on ice before storaging at -80°C for further analysis. DNA extraction method was as previous described (Manichanh et al., 2006). Briefly, a aliquot (200 mg) of each fecal sample was suspended in a mixture of 40 μl 10% *N*-lauroylsarcosine, 0.1 M Tris (pH 7.5) and 250 μl guanidine thiocyanate. DNA integrity and concentration were measured by agarose gel electrophoresis (concentration of agarose gel: 1%; voltage: 150 V; electrophoresis time: 40 min) and nanodrop instrument (Thermo Fisher Scientific), respectively.

### 16S rRNA Gene Amplification and Sequencing

The 16S rRNA gene amplification and sequencing were performed at the lab of BGI-WuHan (Beijing Genomic Institute-WuHan Huada Gene Institute). The amplification of the V4 region (515–806) of the 16S rRNA gene was conducted with universal primer pairs (515F 5′-GTGCCAGCMGCCGCGGTAA-3′ and 806R 5′-GGACTACHVGGGTWTCTAAT-3′), the reverse primer contained unique barcode sequences and appropriate adapters tag each PCR products to distinguish different samples. For library construction, per 50 μl PCR mixture consisted of 30 ng DNA template, 4 μl PCR primer cocktail (515F–806R), 25 μl PCR master mix (NEB Phusion High-Fidelity PCR Master Mix) and appropriate volume of ddH_2_O as need. The PCR program as follows: initial denaturation of 98°C for 3 min, followed by 30 cycles of denaturation at 98°C (45 s), annealing at 55°C (45 s), extension at 72°C (45 s), and the final extension of 72°C for 7 min. All PCR products were purified with Ampure XP beads (AGENCOURT) to remove the unspecific products, the average molecule length and concentration of final amplicon library were estimated using the Agilent 2100 bioanalyzer instrument (Agilent DNA 1000 Reagents) and real-time quantitative PCR (qPCR) (EvaGreen^TM^), respectively. After validation of the library, the qualified libraries were sequenced on the MiSeq platform with the sequencing strategy PE250 following the manufacturer’s instructions.

### 16S rRNA Gene Analysis of Intestinal Flora

High quality reads in subsequent bioinformatics analysis were obtained through in-house pipeline (Huada Gene). In brief, the criteria for collecting clean data from raw data included removal of low quality reads whose mean quality fell below 20 over a 25 bp sliding window based on the Phred algorithm, adapter sequences, ambiguous base (N base), and low complexity reads (default: reads with 10 consecutive same base). The high-quality paired end reads were conjuncted to tags based on overlaps by FLASH (Fast Length Adjustment of Short reads, v1.2.11; Magoč and Salzberg, 2011). Chimeric sequences, detected using UCHIME algorithm (Edgar et al., 2011; Rooks et al., 2014) by against GOLD database, were discarded prior to performing open-reference OTU picking with QIIME (Navas-Molina et al., 2013) v1.9.1 at 97% of sequence similarity. The OTUs whose relative abundance less than 0.005% were discarded to reduce the disturbance of the low abundance spurious OTUs according to Bokulich et al. (2013). The taxonomy assignment was against the GreenGenes database using RDP classifier with the confidence value of at least 0.8, and adopting the PyNAST method (DeSantis et al., 2006a) to align the representative sequences against the GreenGenes core set (DeSantis et al., 2006b). The phylogenetic tree was generated using FastTree prior to the diversity analyses. Alpha diversity analysis (Shannon index, rarefaction analysis) and beta diversity analysis (unweighted UniFrac distance) were calculated using QIIME. Principal coordinates analysis (PCoA) and hierarchical clustering were different forms that visualized the beta diversity. All diversity measurements were conducted on OTU tables rarefied to 25,393 sequences per sample. Potential Microbial biomarkers associated with particular interventions were identified through LEfSe^1^ (Linear Discriminant Analysis Effect Size) with effect size threshold of 2 (Segata et al., 2011). PICRUSt^2^ (Phylogenetic Investigation of Communities by Reconstruction of Unobserved States) was against the GreenGenes database with default settings (type of functional predictions: KEGG Orthologs) to predict the functional profiling of microbial communities based on the 16S rDNA sequences (Langille et al., 2013). STAMP^3^ was used for functional profiling (Parks et al., 2014).

### Whole-Metagenome Shotgun Sequence Analysis

The extracted DNA from the control group, the model group, and the SASP group (*n* = 6, average 2 per group) were sequenced on the Hiseq 4000 Sequencer (Hiseq 4000 SBS Kit, Illumina) with the read lengths 150 bp and insert size of the DNA fragments 350 bp according to the manufacturer’s directions by Huada Gene Institute. The pre-process of the sequencing data was similar to the 16S rRNA gene processing, including the filtration of adapter sequences, low quality reads, and ambiguous base. To exclude the potential host contaminations, quality filtered sequences that aligned to the Rats reference genome (rn5) were removed using the BWA (Burrows–Wheeler Aligner) algorithm with default parameters (Li and Durbin, 2010). All the high-quality sequences of the six samples were assembled by SOAPdenovo2 (Short Oligonucleotide Analysis Package; parameters: -d 1 -M 3 -R -u -F) (Luo et al., 2012). For each sample, we used a series of *k*-mer values (from 49 to 87) and chose the optimal one with the longest N50 value for the remaining scaffolds (Qin et al., 2014). We mapped the clean data against scaffolds using SOAP2 (Version 2.21, parameters: -u, -2, -m 200; Qin et al., 2014). Unused reads from each sample were assembled using the same parameters (Karlsson et al., 2013). Genes (minimum length of 100 nucleotides) were predicted on scaftigs (i.e., continuous sequences within scaffolds) longer than 500 bp using MetaGeneMark v2.7. Then, a non-redundant gene catalog was constructed with CD-HIT (version 4.5.8, parameters: -G 0 -aS 0.9 -g 1 -d 0 -c 0.95) using a sequence identity cut-off of 0.95, with a minimum coverage cut-off of 0.9 for the shorter sequences (Li and Godzik, 2006). To determine the abundance of genes, the high quality reads from each sample were aligned against the gene catalog by SOAP2 (parameters: -m 200 -x 400 identity ≥95%; Li et al., 2014; Qin et al., 2014). Only genes with ≥2 mapped reads were remained in a sample (Qin et al., 2010). The abundance of genes was calculated by counting the number of reads and normalizing by gene length (Qin et al., 2012). BLASTP (Altschul et al., 1990) was used to search the protein sequences of the predicted genes within the KEGG database (Kanehisa et al., 2008) with *E* ≤ 1e-5. The genes were annotated using the KEGG homologs with the lowest e-value. Each protein was allocated to KO (KEGG Orthology group) based on the highest scoring hits with at least one HSP > 60 bits (Backhed et al., 2015). The abundance of KO was calculated by summing the abundance of genes annotated to the same feature.

### Statistical Analysis

Significant *P*-values associated with microbial clades identified by LEfSe were corrected for multiple hypotheses testing using the Benjamini–Hochberg FDR method. Significant *P*-values associated with microbial functions were performed by STAMP, using one-way ANOVA followed by Tukey–Kramer *post hoc* test, Benjamini–Hochberg FDR method was used to correct multiple comparisons. Other statistical tests for significance were performed in GraphPad Prism version 5 for windows.

### Sequence Accession Numbers

The sequences generated in the present study are available through the NCBI Sequence Read Archive (accession number SRP107031).

## Results

### SASP Reduces Inflammation in Rats with TNBS-Induced Colitis

**FIGURE 1:**
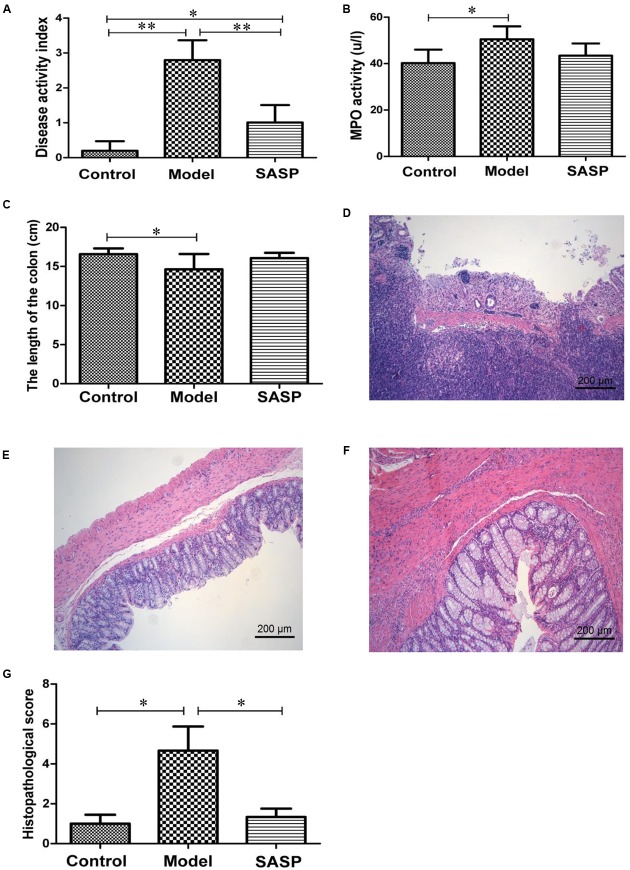
SASP reduces inflammation in TNBS-induced colitis. Effects of SASP treatment on the disease activity index **(A)**, MPO activity **(B)**, the length of the colon **(C)**, histopathological score **(G)**. And the representative pathological section of the colon tissues from rats in the model group **(D)**, the control group **(E)**, and the SASP group **(F)**. Scale bar, 200 μm. Data are expressed as mean ± SD. The differences in **(A–C,G)** were analyzed using one-way ANOVA followed by Tukey’s *post hoc* tests (^∗^*P* < 0.05, ^∗∗^*P* < 0.01).

The DAI, a widely accepted indicator that quantifying the symptoms of animal with IBD (Alex et al., 2009), was developed by Cooper et al. (1993). The DAI was calculated by assigning validated and well-established scores for parameters that in analogy with the clinical symptoms of human IBD (Supplementary Table S1). According to the **Figure 1A**, compared with the control group and the SASP group, the model group showed a significantly higher DAI, demonstrating a distinct symptom of CD. The DAI of the SASP group was significantly lower than that of the model group while it was higher than the control group, implying that colitis with SASP treatment could attenuate inflammation.

MPO activity is positively correlated with the neutrophil inflammatory infiltration (Higa et al., 1997). Inflammatory cell infiltration in serum was estimated by the determination of MPO with ELISA. As shown in **Figure 1B**, the MPO activity was higher in model group compared with the control group and the SASP group. Meanwhile statistically significant difference was observed between the model group and the control group, suggesting that there was a bit serious neutrophil inflammatory infiltration in the model group. The SASP group showed modest improvements of MPO activity vs. the model group.

After the dissection, the colon tissue samples were obtained, and the lengths were recorded. We found that the colon length of the model group was generally shorter than the control group and the SASP group (**Figure 1C**). The colon pathological sections of the model group showed mucosal ulceration, inflammatory cell infiltration, moderately diffuse edema, crypt, and surface epithelium lost (**Figure 1D**). The severity of inflammation in control group and SASP group was graded as mild mucosal inflammation (**Figures 1E,F**). Furthermore, as shown in **Figure 1G**, histopathological score indicated that the histological severity of colitis was severer in the model group compared with the control group and the SASP group. Collectively, it was conclude that the TNBS-induced colitis shared similar symptoms with CD and SASP could alleviate inflammation in rats with colitis vs. the rats in the model group.

### Overall Structure Modulation of Intestinal Flora with SASP

**FIGURE 2:**
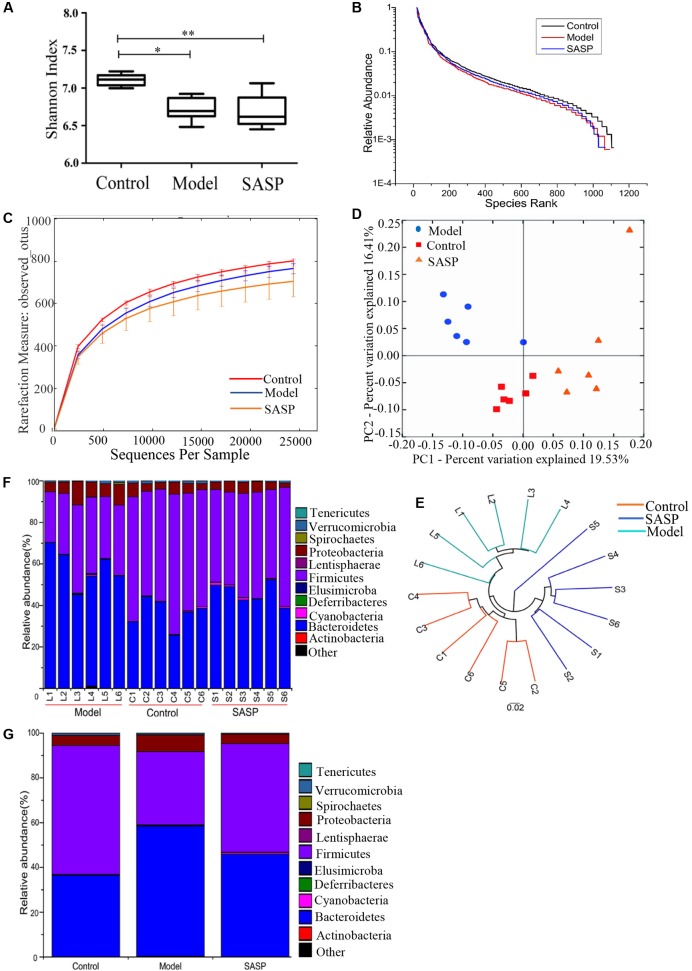
Structural comparison of fecal microbiota among the control group, the model group, and the SASP group. **(A)** The Shannon index was used to estimate diversity of the fecal microbiota among the three groups (data expressed as mean ± SD). **(B)** Rank abundance curve of bacterial OTUs among the three groups. **(C)** The rarefaction analyses-observed_otus could be used to estimate the sequencing depth whether covered rare new phylotypes. **(D)** Plots shown were generated using the unweighted UniFrac-based PCoA. **(E)** Multivariate analysis of variance from unweighted UniFrac-based PCoA matrix scores. Bacterial taxonomic profiling in the phylum level of gut microbiota at individual level **(F)** and different groups level **(G)**. The differences in **(A)** was analyzed using one-way ANOVA followed by Tukey’s *post hoc* tests (^∗^*P* < 0.05, ^∗∗^*P* < 0.01).

The impact of SASP on intestinal flora composition was examined by analyzing bacterial 16S rRNA (V4 region) with MiSeq platform. After removing unqualified sequences (see Materials and Methods), a total of 593,197 raw reads and an average of 32,955 ± 835 reads per sample were obtained, the average length was 252 bp. Following chimera checking, a total of 545,252 effective reads, with an average of 30,292 ± 1271 per sample, remained for downstream analysis. The Shannon indices of the model group and the SASP group were significantly lower than control group (*P* < 0.05 and *P* < 0.01, respectively, **Figure 2A**). As shown in rank-abundance curve (**Figure 2B**), the diversity in descending order is as follow: the control group, the model group, and the SASP group. Meanwhile rarefaction curve analyses already reached stable values at the current sequencing, indicating that the sequencing depth covered rare new phylotypes and most diversity could be captured (**Figure 2C**). Meanwhile, as shown in **Figure 2C**, the value of observed OTUs in the SASP group was significantly lower than the control group (*P* < 0.05). Also, it was less than the model group, indicating that the diversity of the SASP group decreased compared with the other two groups. Generally, combined with the results of Shannon, rank-abundance curve and rarefaction curve analyses, we could find that the biodiversity of the model group was decreased compared with the control group. Furthermore, by contrast with the other two groups, the biodiversity of the SASP group was decreased.

UniFrac-based PCoA displayed an obvious clustering of microbiota composition for each treatment group (**Figure 2D**). The analysis of similarity (ANOSIM) test using Bray–Curtis dissimilarity showed that the observed cluster patterns were significant (*R* = 0.6486, *P* = 0.001). Multivariate analysis of variance of unweighted UniFrac metrics revealed a statistically significant separation among the three groups (**Figure 2E**). As shown in the taxonomic profiling (**Figures 2F,G**), compared with the control group and the SASP group, there was a lower proportion of Firmicutes and an increase in Proteobacteria and Bacteroidetes in the model group (**Table 1**). The taxonomic profiling demonstrated that SASP treatment could increase the level of Firmicutes and reduce the level of Proteobacteria and Bacteroidetes, modulating the gut composition of rats with experimental colitis to levels similar to that of the control group.

**Table 1:** The differences in relative abundance (% ± SD) of four major bacterial phyla among the different three groups.

Major phyla	Relative abundance (%)			*P*-value^∗^		
	Control	Model	SASP	Model vs. control	Model vs. SASP	Control vs. SASP
Bacteroidetes	36.48 ± 6.75	58.04 ± 9.09	45.87 ± 5.23	0	0.026	0.094
Firmicutes	57.52 ± 5.88	32.76 ± 6.36	48.49 ± 5.50	0	0.001	0.046
Proteobacteria	4.49 ± 1.48	7.43 ± 2.68	4.22 ± 1.29	0.045	0.028	0.968
Tenericutes	0.48 ± 0.29	0.59 ± 0.38	1.01 ± 0.29	0.222	0.043	0.031

^∗^*P-values were calculated using Tukey’s honest significant difference test. *P*-values <0.05 were considered statistically significant*.

### Key Phylotypes Responding to the SASP Treatment in TNBS-Induced Colitis

**FIGURE 3:**
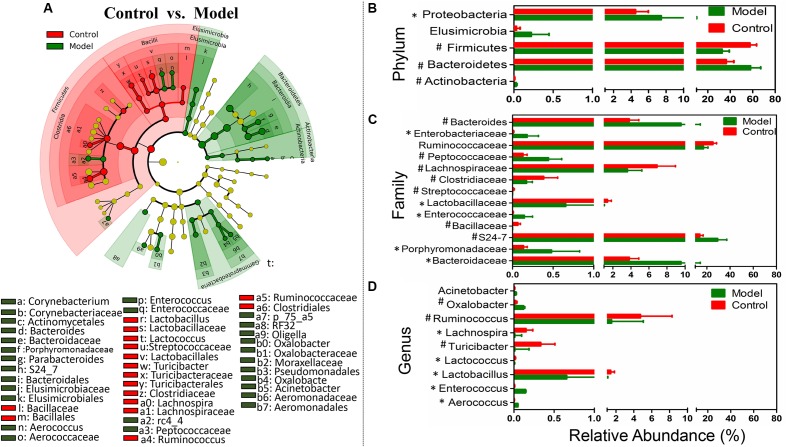
Taxonomic differences of fecal microbiota between the control group and the model group. **(A)** Differentially abundant microbial cladogram obtained by LEfSe, the brightness of each dot is proportional to its effect size. Comparison of relative abundance at the phylum **(B)**, family **(C)**, and genus **(D)** levels between the control group and the model group; all the differences were analyzed using Mann–Whitney *U* test; ^∗^*P* < 0.05; ^#^*P* < 0.01.

We used LEfSe to identify the specific bacterial phylotypes that were altered by TNBS administration and SASP treatment. To investigate the effects of the experimental colitis acting on the microbiome, the differentiated taxon between the control group and the model group was detected. The cladogram representative of the structure and their dominant bacteria were shown in **Figure 3A** and Supplementary Figure S1. Differential microbial lineages for the control group included the Firmicutes, particularly *Ruminococcus*, *Lachnospira*, *Lactobacillus*, *Lactococcus*, and *Turicibacter*; In contrast, clades associating with the model group included the Elusimicrobia, Elusimicrobiaceae; the Actinobacteria, *Corynebacterium*; the Proteobacteria, particularly *Psychrobacter*, *Acinetobacter*; the Firmicutes, *Enterococcus*, *Aerococcus*; and the Bacteroidetes, *Bacteroides*, and *S24_7*. The genera belonging to the five predominant phyla could be taken as biomarkers to distinguish the two communities. The changes in the microbiota after TNBS administration were also explored using the Mann–Whitney *U* test at different taxon levels including phylum, family, and genus (**Figures 3B–D**). Collectively, compared with the control group, the model group possessed a decreased level of SCFAs-producing bacteria, such as Ruminococcaceae (including *Ruminococcus*) and Lachnospiraceae (including *Lachnospira*) (**Figures 3C,D**). Meanwhile the level of lactic acid-producing bacteria was also decreased in the model group, e.g., Lactobacillaceae (including *Lactobacillus*) and Streptococcaceae (including *Lactococcus*) (**Figures 3C,D**). Whereas Enterobacteriaceae, *Bacteroides*, Enterococcaceae (including *Enterococcus*) and *Acinetobacter* were enriched in the model group (**Figures 3C,D**). Thus, the changes in the microbiota revealed the intestinal dysbiosis involved in the experimental colitis.

**FIGURE 4:**
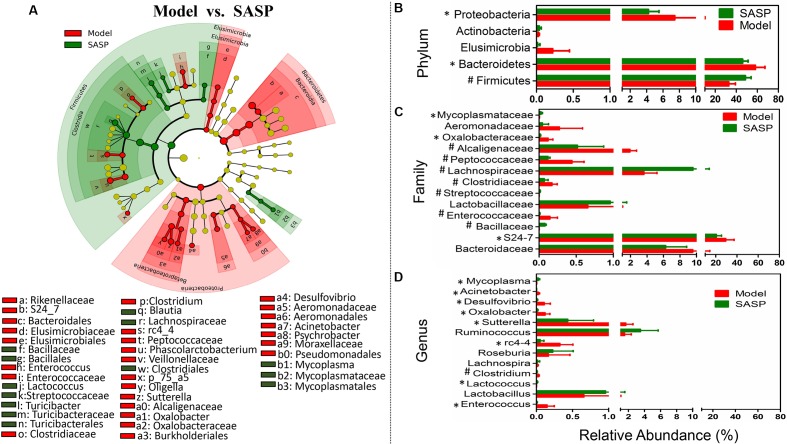
Taxonomic differences of fecal microbiota between the SASP group and the model group. **(A)** Differentially abundant microbial cladogram obtained by LEfSe, the brightness of each dot is proportional to its effect size. Comparison of relative abundance at the phylum **(B)**, family **(C)**, and genus **(D)** levels between the SASP group and the model group; all the differences were analyzed using Mann–Whitney *U* test; ^∗^*P* < 0.05; ^#^*P* < 0.01.

Moreover, to investigate the effects of the SASP treatment on the microbiome, the differentiated taxon between the SASP group and the model group was assessed. **Figure 4A** and Supplementary Figure S2 showed the differences in taxa between the two groups and identified key phylotypes as distinguishing biomarkers at different phylogenetic levels. Differential microbial lineages for the model group included Bacteroidetes, *S24_7*; the Firmicutes, *Enterococcus*, *Phascolarctobacterium*, *rc4-4*; and the Proteobacteria, particularly Desulfovibrionaceae, *Sutterella*, Aeromonadales, *Oligella*, *Psychrobacter*, and *Acinetobacter*. By contrast, clades associated with the SASP group included the Firmicutes, *Blautia*, *Lactococcus*, *Turicibacter*; and the *Tenericutes*, *Mycoplasma*. Meanwhile the changes in the microbiota after SASP treatment were also investigated at different taxon levels including phylum, family, and genus (**Figures 4B–D**). Overall, compared with the model group, there is an increased level of SCFAs-producing bacteria in the SASP group, such as Lachnospiraceae (including *Lachnospira*) and *Ruminococcus* (**Figures 4C,D**). Meanwhile the lactic acid-producing bacteria was also increased, e.g., Lactobacillaceae (including *Lactobacillus*) and Streptococcaceae (including *Lactococcus*) (**Figures 4C,D**). Whereas there was an increased abundance of Enterococcaceae (including *Enterococcus*), *Desulfovibrio*, *Oxalobacter*, and *Acinetobacter* in the model group (**Figures 4C,D**). In addition, the Mycoplasmataceae (including *Mycoplasma*), associated with infertility, were significantly more abundant in the SASP group compared with the model group.

Additionally, to identify gut microbiome responses associated with SASP and legacy effects, we determined the microbial clade differences between the control group and the SASP group using LEfSe (Supplementary Figures S3, S4). Supplementary Figures S3 and S4 showed the differences in taxa between the two groups and identified key phylotypes as distinguishing biomarkers at different phylogenetic levels. Differential microbial lineages for the control group included Firmicutes, particularly *Lachnospira*, and *Clostridium*; in contrast, clades associating with the SASP group included the Bacteroidetes, *Bacteroides*, and *S24_7*; Proteobacteria, *RF32*, Enterobacteriaceae; and *Tenericutes*, *Mycoplasma*. In contrast to the large differences in fecal microbiota between the control group and the model group, only a few microbial signatures in the fecal microbiota were different between the control group and the SASP group.

Collectively, the results showed that an increased level of Enterococcaceae, Proteobacteria (particularly Enterobacteriaceae, *Acinetobacter*, *Oxalobacter*, and *Desulfovibrio*) and a decreased level of lactic acid- and SCFAs-producing bacteria appeared in the model group when compared with the control group and the SASP group. Based on the results among the different three groups, it was discovered that SASP treatment could restore the gut microbiome of rats with experimental colitis to a similar composition of the control group. Moreover, a higher proportion of *Mycoplasma* in the SASP group relative to the model and control groups may be used to explain the side effect of fertility in patients treated with SASP.

### Microbial Metabolic Functions Associated with SASP Treatment in TNBS-Induced Colitis

**FIGURE 5:**
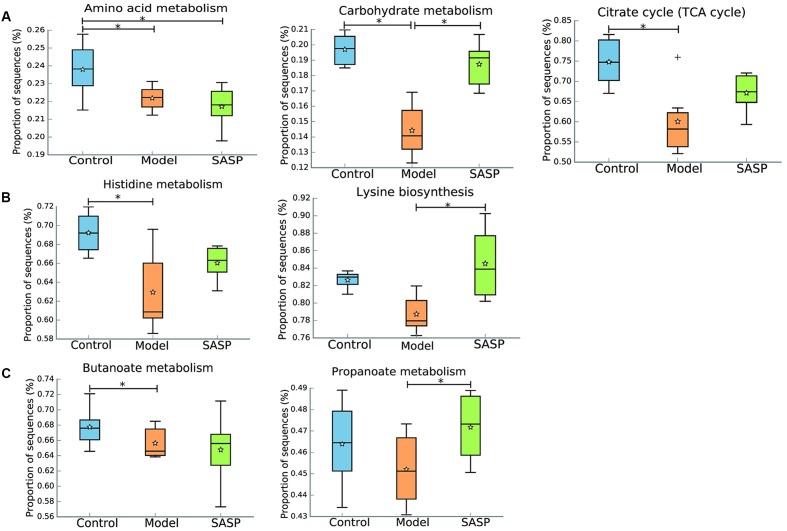
Inferred gut microbiome functions by PICRUSt from 16S rRNA gene sequences among the different three groups (the control group, the model group, and the SASP group). **(A)** Basic metabolism including amino acid metabolism, carbohydrate metabolism, and citrate cycle. **(B)** The metabolism and biosynthesis of amino acids, including histidine metabolism and lysine biosynthesis. **(C)** SCFA production including propanoate and butanoate metabolism. Box plots denote the top quartile, median and bottom quartile, and white stars mean the average value as well as the ‘+’ means the outlier. All the differences were analyzed using one-way ANOVA followed by Tukey–Kramer *post hoc* test, Benjamini–Hochberg FDR method was used to correct multiple comparisons (^∗^*P* < 0.05).

To investigate the gut microbiome functions related to the TNBS administration and SASP treatment, we adopted PICRUSt to infer putative metagenomes from 16S rRNA gene profiles. The summarized information of PICRUSt analysis was shown in **Table 2**. STAMP was used to identify microbially relevant functions linked with the TNBS administration and SASP treatment, and it also can be used to generate PCA (principal components analysis) analysis based on the PICRUSt analysis (KEGG level). PCA revealed an obvious clustering of microbiota composition for each treatment group (Supplementary Figure S5). The ANOSIM test using Bray–Curtis dissimilarity showed that the observed cluster patterns were significant (*R* = 0.6856, *P* = 0.001). The decreased basic metabolisms were presented in the model group when compared with the control group and the SASP group (**Figure 5A**), such as carbohydrate metabolism and citrate cycle (TCA cycle). As regards the amino acid metabolism, there was a significantly decreased in the model group and the SASP group when compared with the control group, and there was no difference between the model group and the SASP group. Meanwhile, genes for the metabolism and biosynthesis of amino acids (specifically lysine and histidine) reduced sharply in the model group (**Figure 5B**). The current situation of basic metabolisms suggested that the gut microbiome in the model group can reduce energy harvest to in favor of nutrient uptake compared with the control group and the SASP group. Moreover, a potential decrease in propanoate and butanoate metabolism in the model group was found by comparing with the control group and the SASP group (**Figure 5C**), indicating a potential decrease in SCFA production, which possibly due to the reduction of SCFAs-producing bacteria, e.g., Ruminococcaceae, Lachnospiraceae, and *Blautia*.

**Table 2:** Summary of PICRUSt analysis.

	Control	Model	SASP
Mapped sequences (% total)	20,171 ± 971	20,477 ± 1124	18,523 ± 1646
	(79.6 ± 2.9)	(73.6 ± 3.7)	(74.2 ± 7.1)
Reference-based OTUs	419 ± 11	410 ± 13	372 ± 36
Weighted NSTI	0.161 ± 0.007	0.183 ± 0.021	0.174 ± 0.021
KOs	14,228,688 ± 1,249,910	11,740,836 ± 1,350,269	12,815,535 ± 1,084,953

*Number and percentage of the 16S rRNA gene sequences that mapped to GreenGenes database at 97% similarity; the number of reference-based OTUs; the weighted NSTI (Nearest Sequenced Taxon Index) value representing the accuracy of metagenome prediction (lower values imply a more accurate prediction); and the number of inferred KOs (KEGG Orthology groups) for stool samples (mean ± SD). (Note: non-microbial categories, e.g., “Organismal Systems” and “Human Diseases,” were excluded from further analysis.)*

**FIGURE 6:**
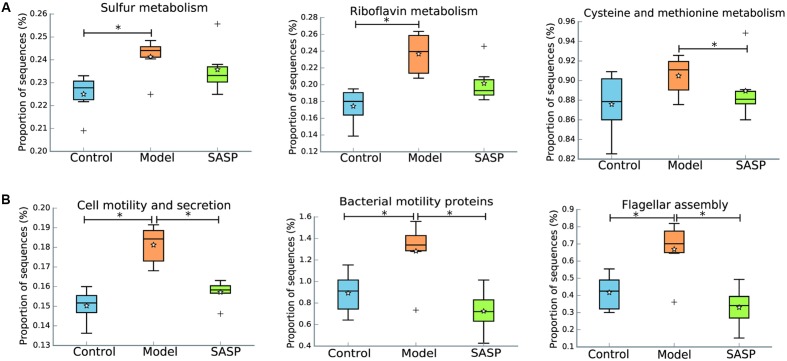
Inferred gut microbiome functions by PICRUSt from 16S rRNA gene sequences among the different three groups (the control group, the model group, and the SASP group). **(A)** Biosynthesis of compounds for oxidative stress including riboflavin metabolism, sulfur metabolism, cysteine and methionine metabolism. **(B)** Dysregulated cellular processing pathways were presented in the model group, including cell motility and secretion, bacterial motility proteins and flagellar assembly. Box plots denote the top quartile, median and bottom quartile, and white stars mean the average value as well as the ‘+’ means the outlier. All the differences were analyzed using one-way ANOVA followed by Tukey–Kramer *post hoc* test, Benjamini–Hochberg FDR method was used to correct multiple comparisons (^∗^*P* < 0.05).

The IBD metagenome has an enhanced capacity for managing oxidative stress, a feature of the inflammatory environment, as indicated by incremental riboflavin and sulfur metabolism, cysteine and methionine metabolism in the model group by comparing with the control group and the SASP group (**Figure 6A**). The increased levels of riboflavin and sulfur metabolism, cysteine and methionine metabolism could indicate that the biosynthesis of advantageous compounds for oxidative stress were increased in the model group. The increases in those compounds may reflect a shift toward an inflammation-promoting microbiome.

Additionally, dysregulated cellular processing pathways were also presented in the model group when compared with the other two groups, including cell motility and secretion, bacterial motility proteins and flagellar assembly (**Figure 6B**). Flagellar bacterial antigens have been involved in both human IBD patients and mouse models of colitis along with the disease development, such as the Cbir1 flagellin (Lodes et al., 2004). All in all, the results demonstrated that TNBS administration result in gut dysfunction and indicated that SASP treatment could modulate the gut microbiome functions of the TNBS-induced colitis model to a similar level of the control group.

**FIGURE 7:**
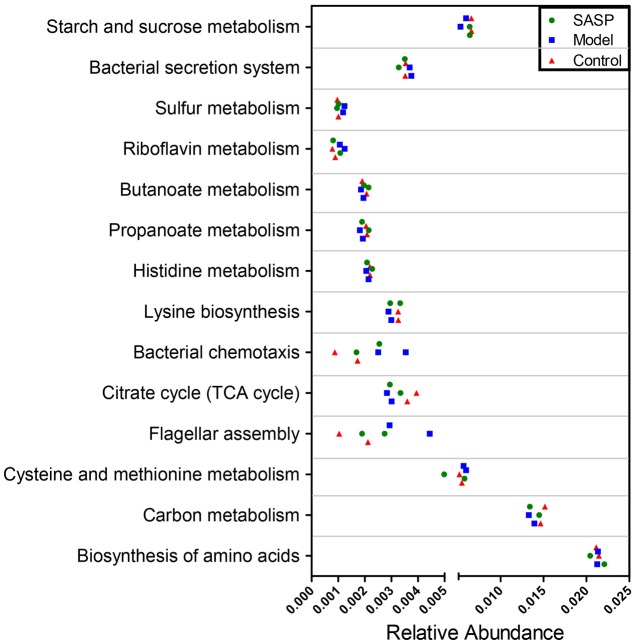
Shotgun sequencing validates the predicted microbial metabolic trends in a small subset of fecal samples from the different three groups (the control group, the model group, and the SASP group). A small subset of six stool samples were subjected to shotgun metagenomic sequencing via Hiseq 4000 platform. Of the 14 microbial metabolic modules, 11 retained the same under- or over-abundance trend predicted from 16S gene sequencing, excluding the biosynthesis of amino acids.

We validated the inferred functions using shotgun metagenomic sequencing based on a subset of six fecal samples from the different groups (**Figure 7**). A total of 238,376,348 qualified shotgun sequences were obtained with an average of 39,729,391 ± 476,817 reads per sample. As shown in **Figure 7**, 11 metabolic modules retained the same over- or under-abundance trend predicted from 16S rRNA gene sequencing, excluding biosynthesis of amino acids and butanoate as well as histidine metabolism. Insight into these functional variations was illuminated in Supplementary Table S2. Briefly, genes involved in bacterial chemotaxis, flagellar assembly, and sulfur metabolism were over-represented in the microbiomes of the model group compared with the other two groups. On the contrary, genes related to the basic metabolism (e.g., TCA cycle, propanoate and butanoate metabolism) were underrepresented.

One of the most striking observations was that genes involved in flagellar assembly were significantly over-represented in the microbiome of the model group (Supplementary Table S2). Compared with the control group and the SASP group, there was an increase in synthetic genes encoding the hook (FliE and FlgE genes) of flagella. Encoding genes of FliH/S/T/G/M/N, also had a higher abundance in the model group.

An over-representation of sulfur metabolism-related genes was observed in the microbiome of the model group (Supplementary Table S2). For example, genes related to sulfate transport (cysU/W/A) were overrepresented in the model group compared with the other two groups. In addition, genes involved in bacterial chemotaxis were significantly overrepresented in the microbiome of the model group, e.g., chemotaxis histidine protein kinase (CheA) gene and response regulator CheB (Supplementary Table S2).

Additionally, there was a decrease of genes related to basic metabolism (e.g., TCA, propanoate and butanoate metabolism) in microbiomes of the model group (Supplementary Table S2). For example, encoding genes of enzymes that mediate TCA cycle and propanoate metabolism as well as butanoate metabolism were under-represented in the model group. These enzymes include acnB, which catalyzes the transformation between citric acid and citric acid, and DLD, which involved in the process of 2-ketoglutaric acid into succinyl-CoA, and aarC/cat1, which catalyzes succinyl-CoA into succinic acid, and mqo, which catalyzes malic acid into oxaloacetic acid, as well as mcmA2/1, which catalyzes methylmalonyl-CoA into succinyl-CoA.

## Discussion

The mechanism of colitis initiation mainly includes spontaneous colitis in certain inbred mouse, chemical-induced colitis (e.g., TNBS, DSS, and acetic acid), and genetically engineered animal model (Mizoguchi, 2012). The most widely used animal models are induced by administering chemicals. Most of animal models showed rapid self-healing ability in the colon, which diverged from the chronic characteristic of human CD. A previous study indicated that the symptoms of IBD significantly lasted longer in the second cycles of chemicals administration (Gaudio et al., 1999). Thus, in the present study, two cycles of TNBS administration method (once a week for 2 weeks) was used to induce colitis. The DAI, MPO, histopathological score, and pathological sections of the model group proved that the Sprague-Dawley rats which clystered with TNBS developed similar symptoms as the human CD (**Figure 1**). Meanwhile, compared with the model group, the results of the SASP group indicated that SASP treatment could alleviate the inflammation (**Figure 1**).

Currently, the known number of uncultured phylotypes has been estimated to add up to about 1800, and the microorganisms of the intestinal tract approximately 10 times more numerous than cells (Zoetendal et al., 2008). Owing to its complexity, it is difficult to comprehensively describe the intestinal microbiota through the traditional microbiological techniques. Recently, the high-throughput sequencing techniques set a stage for the development of IBD. Rarefaction curve analyses already reached stable values in the present study, indicating that the current sequencing has satisfied the most diversity and covered rare new phylotypes (**Figure 1C**). Meanwhile a decrease of the alpha diversity was observed in the model group compared with the control group (**Figures 1A–C**). This case implied that a decrease in biodiversity in the model group. A previous study showed that the dwindling of diversity was associated with a declining diversity of the Firmicutes (Kang et al., 2010), and has also been linked with transitory instability in the dominant taxa in both CD and UC (Martinez et al., 2008). Furthermore, there was a declining alpha diversity in the SASP group compared with the control group and the model group, suggesting that the abundance of gut microbiome in rats with SASP treatment would further decrease. The case might associate with the high concentration of sulfonamide released in the colon, which caused an antibacterial effect.

Many studies found that structural dysbioses occurs in IBD. Although there are no consistent opinion from experts or definitive evidence existed to prove that what microorganisms are the cause of CD. Some experimental results have shown the same changing tendency of some microbiota, including the decrease of several representative taxa within the Firmicutes phylum and the increase of the Gammaproteobacteria class (Sokol and Seksik, 2010). In the current study (**Figures 2F,G** and **Table 1**), a lower percentage of Firmicutes phylum and an increasing number of Proteobacteria phylum and Bacteroidetes phylum have appeared in the model group when compared with the control group and the SASP group. Intriguingly, the number of Bacteroidetes decreased in CD as previous reported (Kostic et al., 2014), but the present study just has the opposite changing tendency, which was consistent with a previous study using TNBS-induced colitis model (He et al., 2016). So the TNBS-induced colitis method may affect the bacterial changing tendency in some ways.

The effects of the experimental colitis acting on the microbiome were investigated by comparing control group and the model group (**Figure 3**). The gut microbiota of the model group is associated with the decreased levels of Firmicutes, including Ruminococcaceae and *Lachnospira*. The Ruminococcaceae, which is a producer of acetate and butyrate, involved in the first step of microbiome-associated carbohydrate metabolism, degrading several types of polysaccharides in the intestinal tract (Flint et al., 2008). Interestingly, the Ruminococcaceae would further consume hydrogen and produce acetate that can be utilized by *Roseburia* to produce butyrate (Chassard and Bernalier-Donadille, 2006). The *Lachnospira* also are the producers of acetate and butyrate (Ling et al., 2014). The low abundance of Ruminococcaceae and *Lachnospira* in the model group means that the SCFAs production were reduced. This corresponded with an underrepresentation of genes related to propanoate and butanoate metabolism, such as DLD, mcmA2/1, acnB, and aarC/cat1 (Supplementary Table S2). SCFAs include acetate, propionate, and butyrate, products of gut microbiota ferment dietary fiber. And the SCFAs also are the major energy source for the colonic epithelial cells and recently were reported to improve colonization resistance and suppress pathogen by lower the redox potential (eH) and pH in the intestinal tract (Ahmad et al., 2000; Roberfroid et al., 2010). Thus the decrease of Ruminococcaceae and *Lachnospira* in the model group may affect host inflammation by reducing SCFAs availability. Meanwhile a decrease of the lactic acid-producing bacteria (including *Lactobacillus* and *Lactococcus*) was also observed in the model group. Previous studies indicated that lactic acid-producing bacteria may protect the host from inflammation by downregulating inflammatory signals. Particularly *Lactobacillus* spp. also acted as an ingredient of probiotics to treat IBD (Veerappan et al., 2012). But intriguing, previous study indicated that an increase of *Bifidobacterium* and *Lactobacillus* species was observed in patients with active CD (Wang et al., 2014). The tendency of *Lactobacillus* spp. in IBD remains controversial. So *Lactobacillus* spp. should be used as probiotics more cautiously to treat IBD. Moreover, the gut microbiota of the model group is also associated with the increased levels of the Proteobacteria and *Enterococcus*. *Enterococcus* has been identified previously enriched in CD patients. Particularly *Enterococcus faecalis* has emerged as a pathogen in recent decades, especially in nosocomial infections (Cuzon et al., 2008). And some studies reported that *E. faecalis* could induce IBD in interleukin-10 knockout mice (Balish and Warner, 2002). Taken the above into consideration, the results have shown a decrease of SCFAs-producing bacteria and lactic acid-producing bacteria as well as an increase of pathogenic bacteria in the model group. The significant alteration of specific bacteria may be associated with the development of CD.

Meanwhile, the effects of the SASP acting on the microbiome were assessed by comparing the SASP group and the model group (**Figure 4**). The gut microbiota of the SASP group is associated with the increase of the SCFAs-producing bacteria (Lachnospiraceae—*Blautia*), lactic acid-producing bacteria (*Lactococcus*) and the decrease of *Enterococcus* and Proteobacteria. Collectively, it was concluded that SASP treatment could modulate the gut microbiome of rats with colitis to a composition similar to those of the control group. Furthermore, an increase of the *Mycoplasma* was observed in the SASP group. The role of *Mycoplasma* in female urogenital infections have been demonstrated, such as *Mycoplasma hominis* had been isolated and identified (Jamalizadeh Bahaabadi et al., 2014). The genital infection with *Mycoplasma* have destructive effects on reproductive system for causing death of infants and fertility disorders. A number of studies noted that SASP could cause reversible and non-dose-dependent abnormalities of sperm in most of men (>80%) (Taffet and Das, 1983). The fertility restored after withdrawal of SASP or choosing a different 5-ASA compound without the sulfapyridine component, such as mesalamine (Chatzinoff et al., 1988). So, the sulfapyridine may have direct toxic effects on reproductive function, but the mechanism is poorly explored. Thus, the increased *Mycoplasma* in the SASP group may provide a clue to explain why infertility would occur in those IBD patients with SASP treatment, and it needs further confirmed through monitoring the *Mycoplasma* changing tendency after withdrawal of SASP.

The under- and over-representation of certain pathways and genes in the model group (**Figures 5**–**7** and Supplementary Table S2), such as the decreased basic metabolism and the increased oxidative stress, would make the microbiota to maintain homeostasis during inflammation. Previous study also showed that there was a decrease in biosynthesis of amino acids and an increase in amino acid transporter genes in IBD (Morgan et al., 2012). Thus, the bacteria usually have a weak capacity to produce their own nutrients under inflammatory state, but rather transport them from the available environment (e.g., sites of inflammation or tissue destruction). Meanwhile genes for metabolism of the sulfur-containing amino acid increased in the model group, like cysteine, a precursor of glutathione. Glutathione, synthesized by Proteobacteria and some Enterococci and Streptococci, allows bacteria to keep homeostasis under oxidative stress (Sherrill and Fahey, 1998). The increased cysteine metabolism was consistent with the increases of the Proteobacteria and *Enterococcus* in the model group (**Figures 3**, **4**). Moreover, the results also demonstrated that a decrease in butanoate and propanoate metabolism genes in the model group (e.g., DLD, mcmA2/1, acnB, and aarC/cat1) (Supplementary Table S2), which was also consistent with the decreases of SCFA-producing Firmicutes clades analyzed in taxonomic profiling studies (**Figures 3**, **4**). The case could reveal that the perturbations in bacterial composition were associated with the gut microbiome function. In all, the functional variation of microbiota among the control group, the model group, and the SASP group may indicate that SASP treatment could modulate the dysregulated metabolism pathways in rats with colitis, restoring the abnormal microbiota function to a normal situation as the control group.

The inferred metagenomes from the 16S data were validated by shotgun metagenomic sequencing, providing a confirmation that they were representative of community functional capability. The limitations of the inferred metagenomes also should be considered. The current 16S sequencing mainly includes bacterial and archaeal genomes, ignoring non-bacterial organisms, like viruses, fungi, and phage. Some studies have noted that non-bacterial organisms may play a significant role in the gastrointestinal disease. For example, norovirus infection is necessary in the development of CD mouse model (Cadwell et al., 2010). As the cost of sequencing falls and computational biology develops, large-scale metagenomic data containing dozens or hundreds of samples will further enhance our ability to find out the functions of relevant gene in microbial communities. Thus, to further exploring the undiscovered world, the multifaceted methods, like the unbiased shotgun sequencing, metatranscriptomic, proteomic, and metabolomic, could be taken into consideration.

In summary, our study aims at discovering the gut microbiome composition and function in animal colitis model with and without SASP. The results demonstrated that the experimental colitis with SASP treatment could alleviate the inflammation extent and restore the dysregulated microbiota composition and function of colitis rats in the model group into a normal conditions as the control group. Meanwhile, the increase of *Mycoplasma* in the SASP group may provide a clue to explain why infertility was more prevalent in IBD patients with SASP treatment than the patients without SASP treatment. This result needs further investigate through monitoring the *Mycoplasma* changing tendency after withdrawal of SASP. Moreover, more samples are necessary for exploring the heterogeneity of microbial communities within individuals.

## Author Contributions

HZ and MC carried out the sample collection, data analysis, and drafted the manuscript. YL and ZL revised the final draft of the paper. YW, LW, MW, and FM performed the technical procedures and participated in the sample collection. QL and ZX, who are the corresponding authors, conceived of the study and helped to draft the manuscript. All authors read and approved the final manuscript.

## Conflict of Interest Statement

The authors declare that the research was conducted in the absence of any commercial or financial relationships that could be construed as a potential conflict of interest. The reviewer MB and handling Editor declared their shared affiliation.

## Footnotes

1 http://huttenhower.sph.harvard.edu/lefse/

2 http://picrust.github.io/picrust/

3 http://kiwi.cs.dal.ca/Software/STAMP
