# Supplementary material for: Modulation of Gut Microbiome Composition and Function in Experimental Colitis Treated with Sulfasalazine
Source: Front Microbiol. 2017 Sep 7;8:1703. doi: 10.3389/fmicb.2017.01703 (PMC5594074; doi:10.3389/fmicb.2017.01703)
Supplement: Supplementary file 1 [file Table_1.docx]

**Table S1.** Criteria for scoring disease activity index

| Score weight loss (%) Stool consistency Occult blood or gross bleeding |
| --- |
| 0 None Normal Negative  1 1-5  Loose stool Negative  2 5-10 Loose stool Hemoccult positive  3 10-15 Diarrhea Hemoccult positive  4 >15 Diarrhea Gross bleeding |

Disease activity index = (combined score of weight loss, stool consistency, and bleeding)/3. Normal stools, shape of pellets; loose stools, pasty stools that does not stick to the anus; diarrhea, liquid stools that sticks to the anus.
