# Supplementary material for: Modulation of Gut Microbiome Composition and Function in Experimental Colitis Treated with Sulfasalazine
Source: Front Microbiol. 2017 Sep 7;8:1703. doi: 10.3389/fmicb.2017.01703 (PMC5594074; doi:10.3389/fmicb.2017.01703)
Supplement: Supplementary file 3 [file Data_Sheet_1.docx]

Supplementary Figures


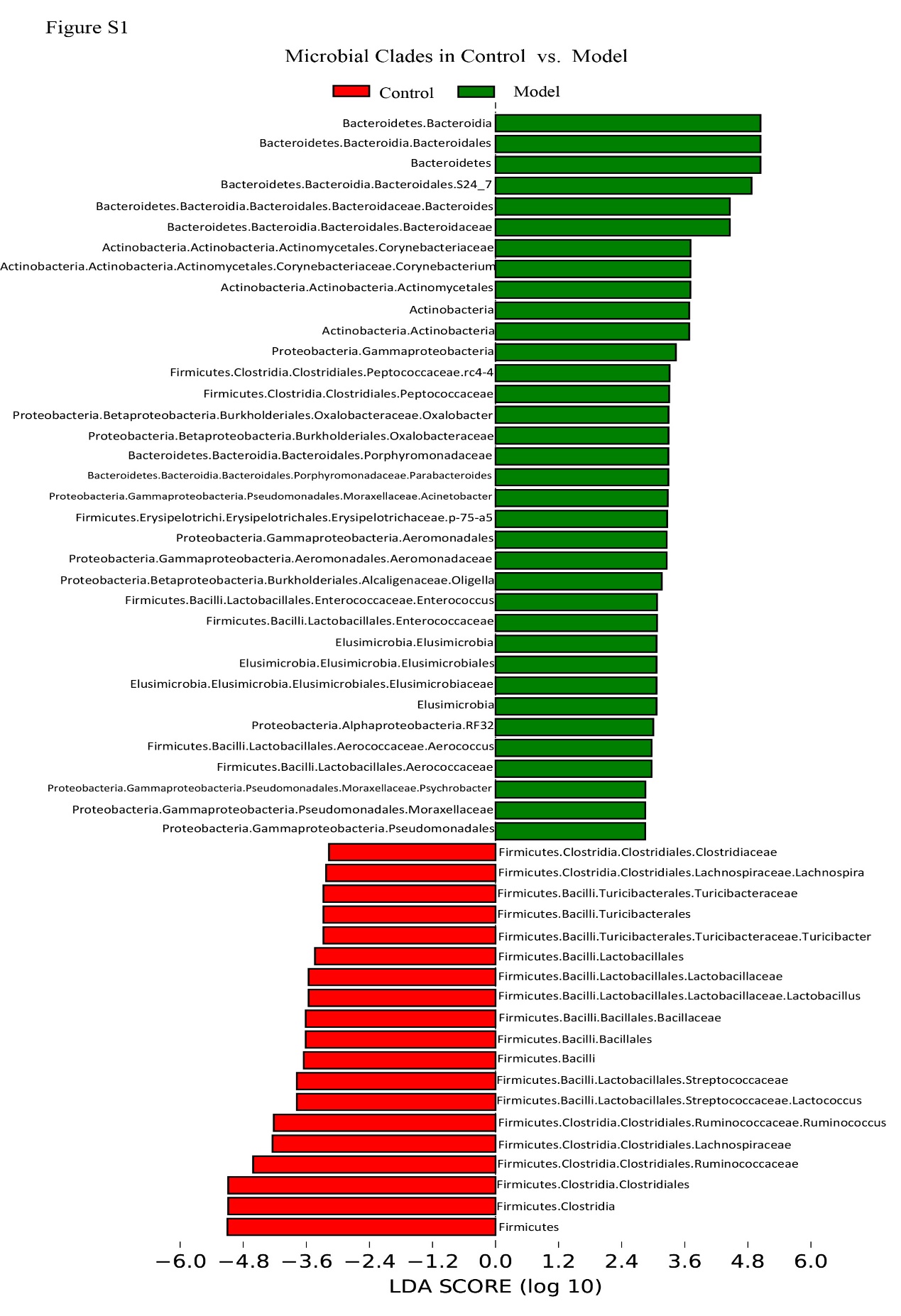


**Figure S1**. LEfSe rank plots of differentially abundant microbial clades in gut microbiome associated with the control group versus the model group. LDA scores for differentially abundant microbial clades in stool from the control group and the model group.


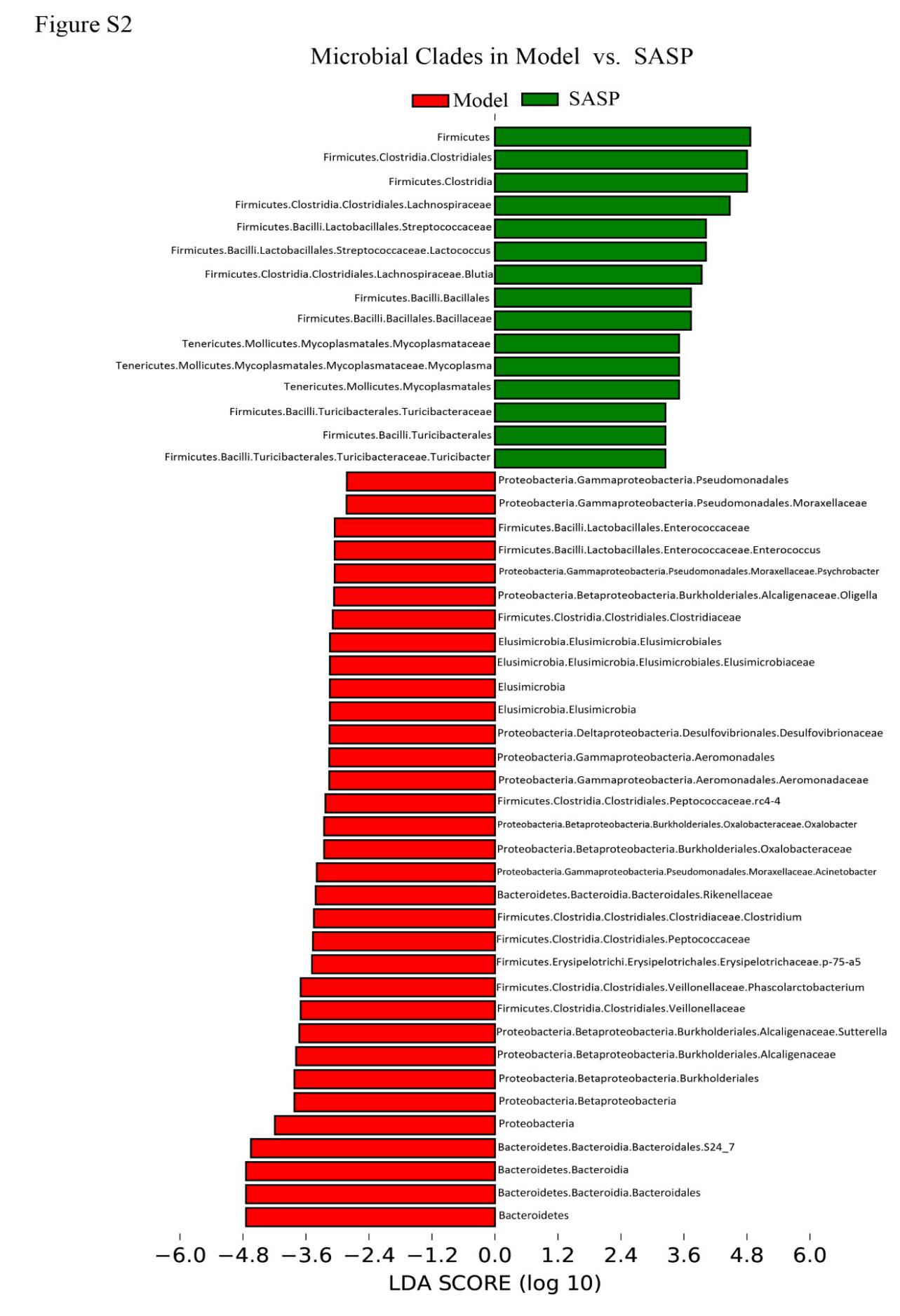


**Figure S2**. LEfSe rank plots of differentially abundant microbial clades in gut microbiomes associated with the SASP group versus the model group. LDA scores for differentially abundant microbial clades in stool from the SASP group and the model group.


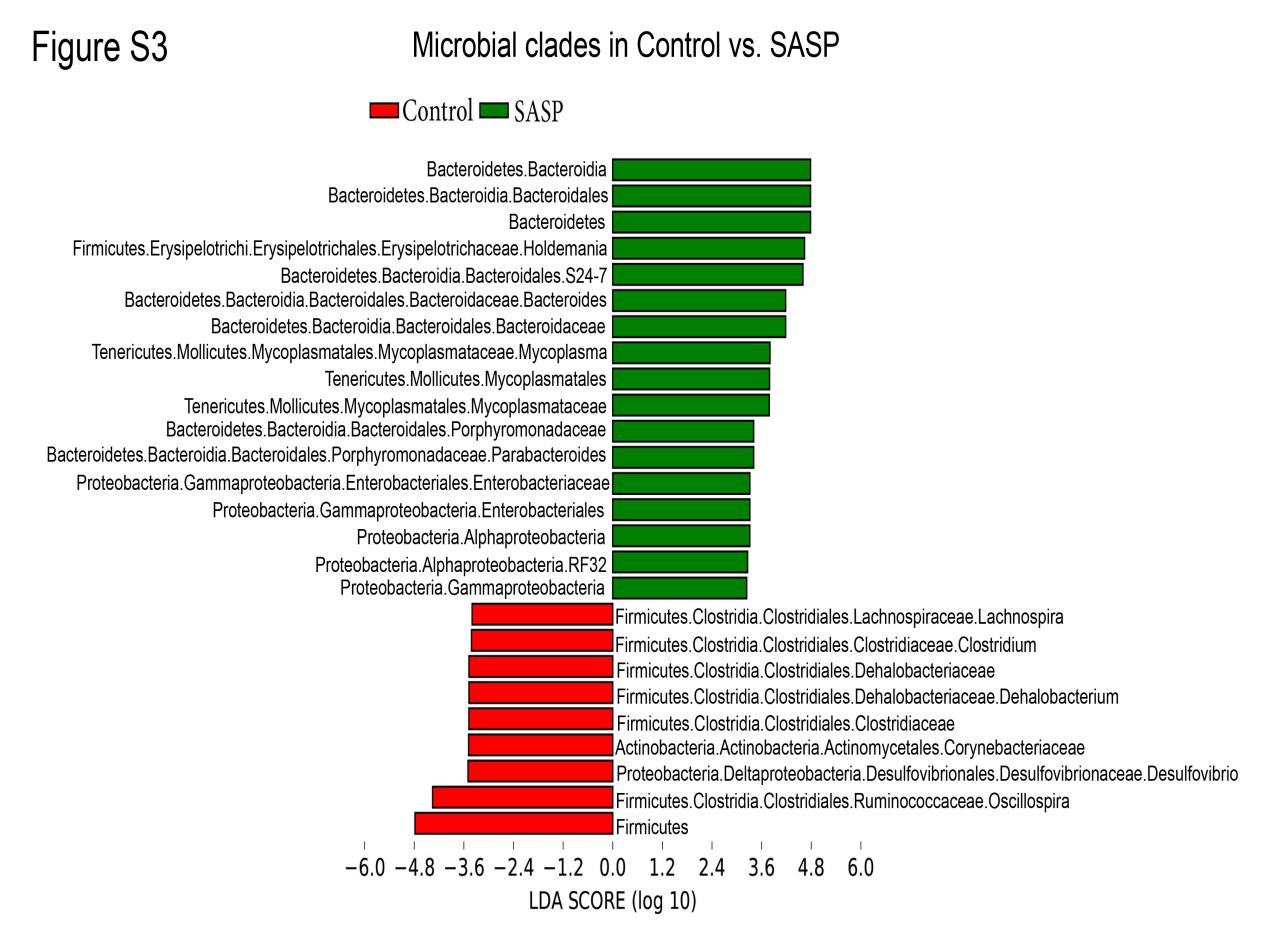


**Figure S3.** LEfSe rank plots of differentially abundant microbial clades in gut microbiomes associated with the SASP group versus the control group. LDA scores for differentially abundant microbial clades in stool from the SASP group and the model group.


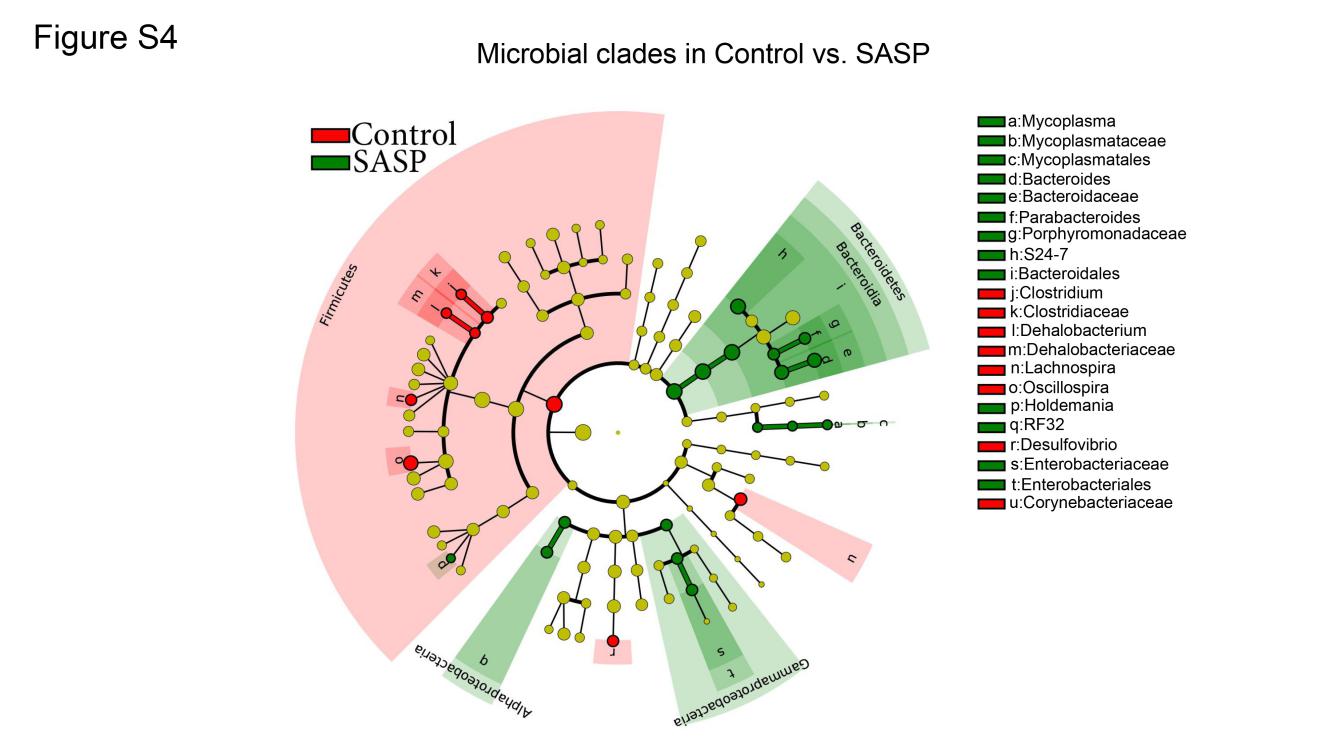


**Figure S4.** Taxonomic differences of fecal microbiota between the SASP group and the control group. Differentially abundant microbial cladogram obtained by LEfSe, the brightness of each dot is proportional to its effect size.


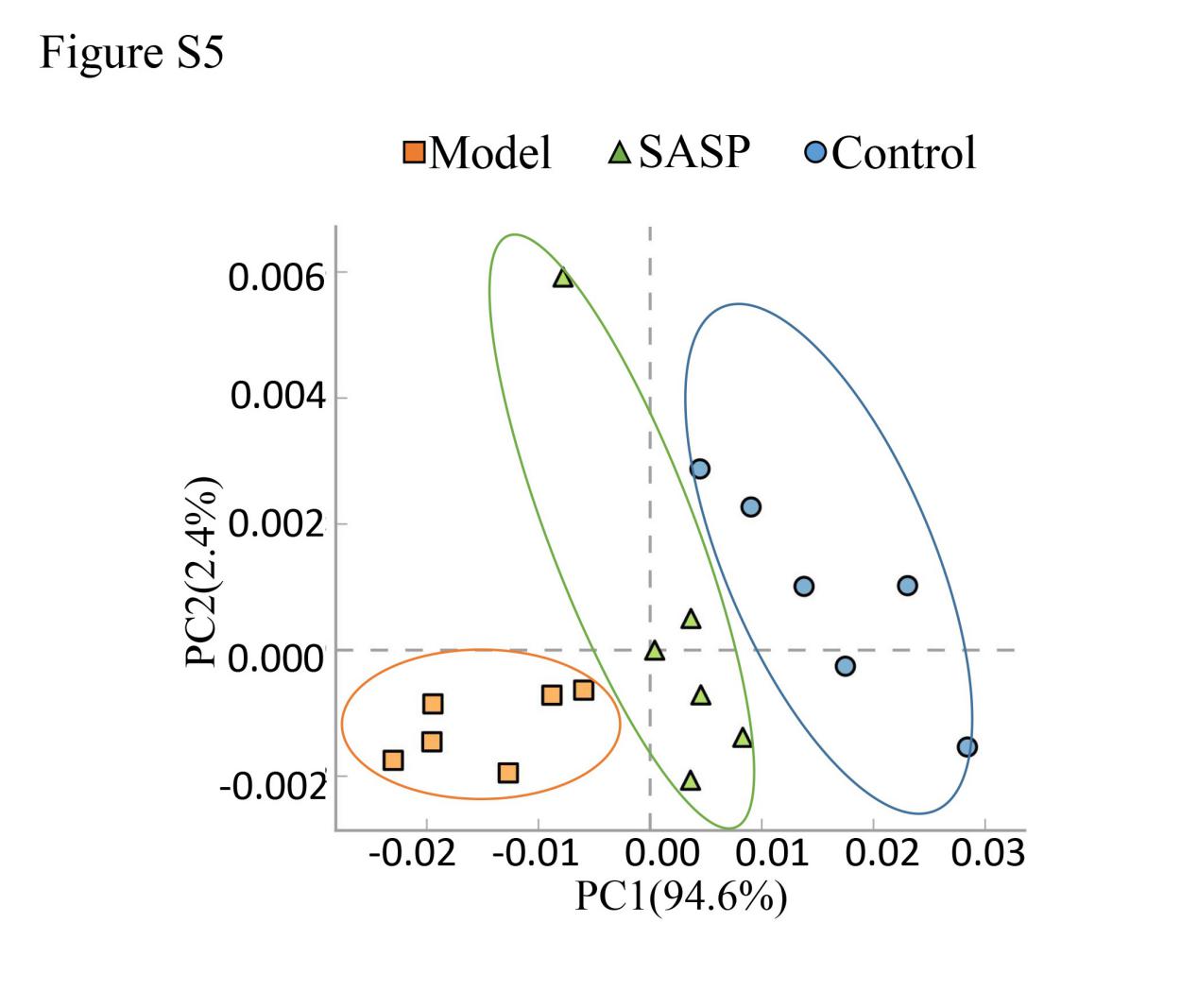


**Figure S5.** PCA at the KEGG level. The PCA of all the samples is based on the annotated KOs’ relative abundance.
